# Mannan-binding lectin serine protease-2 (MASP-2) in human kidney and its relevance for proteolytic activation of the epithelial sodium channel

**DOI:** 10.1038/s41598-022-20213-8

**Published:** 2022-09-24

**Authors:** Rikke Zachar, Steffen Thiel, Søren Hansen, Maiken Lumby Henriksen, Mikkel-Ole Skjoedt, Karsten Skjodt, Zohra Hamzaei, Kirsten Madsen, Lars Lund, Edith Hummler, Per Svenningsen, Boye Lagerbon Jensen

**Affiliations:** 1 Department of Cardiovascular and Renal Research, Institute of Molecular Medicine, University of Southern Denmark, J.B. Winsløvsvej 21, 3, 5000 Odense, Denmark; 2 Department of Biomedicine, Aarhus University, Aarhus, Denmark; 3 Department of Cancer and Inflammation, Institute of Molecular Medicine, University of Southern Denmark, Odense, Denmark; 4 Department of Clinical Immunology, Copenhagen University Hospital, Copenhagen, Denmark; 5 Department of Pathology, Odense University Hospital, Odense, Denmark; 6 Urology and Clinical Department, Odense University Hospital, Odense, Denmark; 7 Department of Biomedical Sciences, University of Lausanne, Lausanne, Switzerland

**Keywords:** Kidney, Proteases

## Abstract

Proteolytic activation of the renal epithelial sodium channel (ENaC) is increased by aldosterone. The aldosterone-sensitive protease remains unidentified. In humans, elevated circulating aldosterone is associated with increased urinary extracellular vesicle (uEVs) excretion of mannan-binding lectin associated serine protease-2 (MASP-2). We hypothesized that MASP-2 is a physiologically relevant ENaC-activating protease. It was confirmed that MASP2 mRNA is abundantly present in liver but not in human and mouse kidneys. Aldosterone-stimulation of murine cortical colleting duct (mCCD) cells did not induce MASP-2 mRNA. In human kidney collecting duct, MASP-2 protein was detected in AQP2-negative/ATP6VB1-positive intercalated cells suggestive of MASP2 protein uptake. Plasma concentration of full-length MASP-2 and the short splice variant MAp19 were not changed in a cross-over intervention study in healthy humans with low (70 mmol/day) versus high (250 mmol/day) Na^+^ intake despite changes in aldosterone. The ratio of MAp19/MASP-2 in plasma was significantly increased with a high Na^+^ diet and the ratio correlated with changes in aldosterone and fractional Na^+^ excretion. MASP-2 was not detected in crude urine or in uEVs. MASP2 activated an amiloride-sensitive current when co-expressed with ENaC in *Xenopus* oocytes, but not when added to the bath solution. In monolayers of collecting duct M1 cells, MASP2 expression did not increase amiloride-sensitive current and in HEK293 cells, MASP-2 did not affect γENaC cleavage. MASP-2 is neither expressed nor co-localized and co-regulated with ENaC in the human kidney or in urine after low Na^+^ intake. MASP-2 does not mediate physiological ENaC cleavage in low salt/high aldosterone settings.

## Introduction

The epithelial Na^+^ channel (ENaC) in the kidney collecting duct principal cells plays an essential role in regulation of renal Na^+^ excretion and blood pressure. The Na^+^-retaining hormone aldosterone induces a shift in the molecular weight of the ENaC γ-subunit, consistent with post-translational proteolytic cleavage of its extracellular loop, leading to its activation^1–4^. Proteolytic activation of ENaC in the apical membrane by cleavage of the extracellular loop contributes both to physiological, Na^+^-sensitive, and aberrant, pathophysiological, regulation of ENaC activity^1,2,5^. Although the increased proteolytical processing of ENaC could be a consequence of increased plasma membrane localization, it is not known whether an aldosterone-sensitive “physiologica” protease is responsible for activation of ENaC.

Several proteases have been shown to activate ENaC in vitro; however, murine gene knockout studies show that the proteases prostasin, kallikrein and TMPRSS4 are dispensable for physiological proteolytic activation^3,6–10^. Urokinase, which is expressed along the nephron and present in urine, fails also to activate ENaC^5,11^. Plasmin is present in minute amounts in urine from healthy individuals and not regulated by aldosterone and is therefore not a likely candidate for physiological regulation, and similar consideration applies for Cathepsin B^12^ and factor VII activating protease (FSAP)^13^. Elevated circulating aldosterone levels, due either to salt-depletion or aldosterone infusion in patients with mild hypertension, increased the excretion of mannan-binding lectin associated serine protease 2 (MASP-2 (protein)/*MASP2* (gene)) in urinary extracellular vesicles (EVs)^14^, which are nano-sized vesicles released from all nephron segments, containing proteins from their parental cell^15^. MASP-2 is necessary for activation of the lectin pathway of the complement system by forming complexes with the soluble pattern recognition molecules (PRMs) mannan-binding lectin (MBL), ficolins (H-, L- and M-ficolins) or collectins (Collectin L1 and K1). MASP-2 is activated when the PRMs bind to carbohydrate residues on microorganisms or “stressed” epithelial cells, leading to potential complement-system-mediated elimination or dysfunction of the target cell^16^.

The urinary excretion of MASP-2 in normal urine samples and its association with uEVs from healthy control persons and essential hypertensive patients was independently confirmed by proteomic analyses^17,18^. Although the identification by mass spectrometry at the protein level confirms MASP-2 protein in urine, its expression in the kidney is controversial. MASP2 mRNA appears to be almost exclusively expressed in the liver and the MASP-2 protein circulates in plasma^19,20^. The molecular weight of the full-length zymogen form of MASP-2 (~ 75 kDa) precludes any significant glomerular filtration in healthy persons. On the other hand, MASP-2 protein has been detected in the human kidney by immunohistochemistry^20^, indicating either local expression or basolateral uptake and apical release leading to urinary excretion. Importantly, the previous studies do not take into account that there is also a splice variant produced from the MASP2 gene, i.e. MAp19, which lacks the catalytical domain but is still able to bind to MBL and ficolin^20^. MAp19 is predominantly expressed in the liver, but the kidneys and thyroid gland have also been detected as sites of expression^20^. While MAp19 has been suggested as a competitive inhibitor of complement activation by MASP-2^21^, this does not appear to be physiologically relevant since the affinity for MAp19 binding to PRMs is much lower than for MASP-2^20^. MAp19 has also been suggested to act as an inhibitor of calcium oxalate kidney stone formation in human urine^22^, and a recent study showed an association between baseline serum MAp19 concentration and the incidence of microalbuminuria in a study cohort of 270 persons with newly diagnosed type 1 diabetes in an 18-year follow up study^23^. Yet, the role of MAp19 in the kidney remains unknown. The present study focused on the observations of increased MASP-2 in human urine by low salt intake^14^ and present a comprehensive characterization of MASP2 in plasma, urine and tissue in humans.

Specifically, the aims of the present study were: (1) to map MASP-2 protein in human kidney tissue, (2) to study its possible regulation in plasma, urine and tissue by low salt/elevated aldosterone in healthy humans and (3) to elucidate its potential to activate ENaC proteolytically. Experiments were designed to test the hypotheses that MASP2 is co-expressed with ENaC in the human kidney collecting duct and its expression is stimulated by aldosterone, and that MASP-2 cleaves and activates γ-ENaC. The hypotheses were tested by application of monoclonal anti-human MASP-2 antibodies to tissue sections in immunohistochemistry and to tissue homogenates, urine and plasma for immunoblotting combined with specific and sensitive ELISAs directed against MASP-2 and MAp19. Moreover, in vitro experiments were done in heterologous expression systems with ENaC to examine effects on ENaC-mediated current by MASP-2 overexpression and MASP-2 addition.

## Methods

### Human kidney and liver tissue

Human kidney tissue was obtained from the Department of Urology, Odense University Hospital from patients undergoing nephrectomy due to renal cancer. As a routine, the patients had fasted 6 h before the operation, and all medicine was discontinued on the day of surgery. The tissue was taken from a non-tumorous part of the kidney and separated into the cortex, outer- and inner medulla 45–60 min after removing the kidney and frozen in liquid nitrogen or fixed in formalin. All patients gave informed written consent; the Ethics Committee of the Region of Southern Denmark (S-20140159) approved the study and the National Data Protection Authority (2012-58-0018) approved the study. The study was conducted according to the declaration of Helsinki. Pools of n = 4 patients were made from the cortex, inner and outer medulla, respectively. Kidney tissue from patients receiving diuretics (n = 3) with predicted high levels of aldosterone were compared to controls receiving no medicine (n = 4). Patient information is shown in Table 1. Anonymized human liver tissue was obtained from patients undergoing liver resection at the Department of Abdominal Surgery, Odense University Hospital. Pools contained samples from 4 patients. The tissue was frozen in liquid nitrogen 45–60 min after removal, homogenized in lysis buffer ((20 mM Tris–HCl, 150 mM NaCl, 20 mM NaF, 10 mM Na_4_P_2_O_7_, 1% Triton X-100) (Merck) with protease inhibitor (C0mplete, Roche, Merck)) and frozen at − 80 °C. Protein concentration was measured with the DC protein assay (BioRad).

**Table 1:** Patient information—human kidney tissue.

Patient category	Patient id (western blot lane number)	Medicine	Gender (male/female)	Age (years)	Blood pressure (mmHg)
Control (no medicine)	1		F	41	139/69
	2		M	45	136/91
	3		M	72	155/83
	4		F	69	150/78
Diuretics (predicted high aldosterone)	1	Thiazide	F	66	190/90
	2	Loop diuretic	F	56	140/90
	3	Thiazide, alpha-blocker, β-blocker, Ca^2+^ antagonist	M	80	115/56

Patient information regarding medicine (pharmacological group), gender, age, and blood pressure from whom kidney tissue was obtained.

*M* male, *F* female.

### Salt intervention samples

Plasma, urine, and uEV samples from a previous study^24^ were included. The included test subjects (n = 10) were given high (250 mmol/day) or low (70 mmol/day) Na^+^ diet for 5 days followed by sample collection and measurement before cross over to the opposite Na^+^ diet. The study was approved by the Ethics Committee of the Region of Southern Denmark (S-20150208), performed in accordance with the Helsinki Declaration, and registered at ClinicalTrials.gov as NCT02823613. All participants gave written informed consent, and the National Data Protection Authority (16/15900) approved the study. Further description of inclusion- and exclusion criteria is outlined in the original paper^24^.

### Urinary extracellular vesicles

Urine from healthy control persons was added protease-inhibitor (Complete, Roche, Merck) and frozen at − 80 °C. The urine was thawed and vortexed for 1 min. Initial centrifugation was performed at 3000*g*, 4 °C for 30 min, and the supernatant was centrifuged at 220,000*g*, at 4 °C for 100 min. The obtained pellet was re-suspended in imidazole buffer (250 mM sucrose, 25 mM imidazole, 1 mM EDTA-Na2; Merck) with protease inhibitor (C0mplete, Roche, Merck), pH = 7.2) in a volume corresponding to 1/1000th of the original volume and frozen at − 80 °C. All patients gave informed written consent, and the Ethics Committee of the Region of Southern Denmark (S-20150208) approved the study, and the project was reported to the National Data Protection Authority (18/31930). When performing western blotting, the uEVs were loaded according to the creatinine concentration of the original urine sample (2.7 μg/well).

### Gel electrophoresis and immunoblotting

The samples were mixed with LDS Sample buffer (NuPAGE, Thermo Fisher Scientific) and reducing agent (NuPAGE) and denatured at 95 °C for 5 min before loading on a 4–12% Bis–Tris gel (NuPAGE). Electrophoresis was performed at 200 V using MOPS-buffer (NuPAGE) and precision Plus Protein Dual Color Standards (Bio-Rad). The proteins were transferred to 0.45 μm Immobilin-P PVDF membranes (Millipore) at 35 V for 1 h in transfer buffer (NuPAGE) with 10% ethanol (Merck). The membrane was blocked with 5% skimmed milk (Merck) in TBST (20 mM Tris-Base, 137 mM NaCl, 0.05% Tween-20 (Merck), pH = 7.6) for 1 h and incubated with primary antibody at 4 °C overnight. An *in-house* mouse monoclonal antibody targeting the SP-domain of MASP-2 (Fig. 2A,B) was used. It was raised against a human MASP-2 peptide aa575-584 LTQRGFLAR coupled to diphteria toxoid and used for immunization, subsequently cloned and selected essentially as previously described^25^. In addition, a monoclonal antibody, targeting the A-chain (clone 1.3B7), previously validated^26^, was also used as primary antibody (Fig. 2A,B). For comparison and validation, a commercial mouse monoclonal antibody targeting the SP-domain of MASP-2 (OriGene Technologies, CAT#: TA812533S) was also applied (Fig. 2A,B). To detect ENaC cleavage in HEK293 cells, rabbit anti-rat gamma ENaC (Stress Marq, SPC-405) diluted 1:1000 was used. Anti-β-actin (Abcam, 8227) was used as a loading control. The membrane was washed with TBST and incubated with HRP-conjugated goat-anti mouse-IgG (0447, Dako), goat-anti rabbit-IgG (P0448, Dako), or rabbit-anti goat-IgG (P0449, Dako) antibody for 1 h. After a subsequent washing step with TBST, ECL (Amersham Bioscience) was applied, and signal developed using ChemiDo XRS + System with Image Lab Software (Bio-Rad). Densitometric analyses were done using Image lab Gel Doc (Bio-Rad). Uncropped original blots are presented in supplementary Fig. S2 and S4.

### Immunohistochemistry and immunofluorescence

Kidney and liver samples were fixed in 4% paraformaldehyde (Merck) and embedded in paraffin. Blocks were cut in 1–4 µm sections, dewaxed, and rehydrated through a series of Tissue-Clear (Sakura ProHosp, Alphen an den Rijn, The Netherlands) and ethanol (99–70%) (Merck). Antigen retrieval was performed with TEG-buffer (10 mM TrisBase, 0.5 mM EGTA Tritiplex VI) (Merck) in a microwave oven for 20 min. Sections were washed in TBST (Merck) and blocked for 30 min in 3% Bovine Serum Albumin (BSA)/TBST (Merck), washed and blocked 10 min in hydrogen peroxide. Anti-MASP-2 (15-17-3) was applied undiluted/in culture supernatant overnight at 4 °C, washed, and incubated with HRP-conjugated goat-anti mouse-IgG (P0447; Dako) diluted 1:1000 for 1 h. Staining was visualized with 3.3′ diaminobenzidine (DAB)(Dako) and counterstained with Mayer's Hematoxylin (Merck). Images were taken with Olympus BX51 microscope with a DP26 camera and Cell Sens software (Olympus, Tokyo, Japan). Co-localization was investigated using anti-MASP-2, anti-AQP2 (collecting duct, principal cell marker, 1:200, 9882, Santa Cruz) and anti-ATP6V1B1 (collecting duct, intercalated cell marker, HPA031847, 1:50), respectively. AlexaFluor488 donkey-anti goat-IgG (Invitrogen) and AlexaFluor555 donkey-anti mouse-IgG (Invitrogen) were used as secondary antibodies.

### Electrophysiological measurements in *Xenopus* oocytes

Purified cRNA was injected into stage V/VI oocytes isolated from *Xenopus laevis* (Noerdhoek, South Africa) as previously described^27^. cRNA (0.25 ng), either coding for human or rat ENaC α, β and γ-ENaC were injected 24 h after harvesting, and oocytes were kept in MBS buffer (87.4 mM Na, 1 mM KCl, 86 mM NaHCO_3_, 2.4 mM HCO_3,_ 0.8 mM MgSO, pH = 7). Electrophysiological measurements were performed 48 h after injection. The oocytes were incubated in Frog Ringer solution (120 mM NaCl (or 50 mM as indicated), 2.5 mM KCl, 1.8 mM CaCl_2_⋅2H_2_O, 10 mM HEPES buffer, (Sigma-Aldrich) pH = 7.2) with different concentrations of the MASP-2 protein for 20–30 min before electrophysiological measurements. Amiloride-sensitive Na current (I_Na_) was recorded using a two-electrode voltage clamp, clamped at − 80 V, defined as the difference between Na^+^ current before and after amiloride. Trypsin was added to fully activate ENaC. Non-injected oocytes were included as negative controls in each experiment. Co-injection experiments were performed where cDNA encoding MASP-2 was subcloned into pSD5 expression vector and in vitro transcribed into cRNA. *Xenopus* oocytes were injected with different cRNA coding concentrations for MASP-2 together with human αβγENaC and incubated in MBS buffer for 24 h before electrophysiological measurements, as described above. Each figure represents a different batch of oocytes.

### Electrophysiological measurement in M1 cells

M1 cells were cultured in Dulbecco's modified Eagle's medium (DMEM)/F12 1:1 (Invitrogen) supplemented with 10% fetal calve serum (FCS, Invitrogen) and pen-strep (Sigma) at 5% CO_2_ at 37 °C. The media was changed every second day. Transfection with plasmid encoding MASP-2 was performed using Metafectene (Biontex, München, Germany). Non-transfected cells were included as controls. The cells were seeded onto permeable filter supports (pore size 0.4 μm, surface area 4.67 cm^2^, Corning Costar), and transepithelial voltage (Vte) and resistance (Rte) were measured using an epithelial Voltohmmeter (EVOM) (World Precision Instruments, Sarasota, FL, USA) on day 1–16 after transferring. On the last day of the experiments, 1 µl of 50 µM amiloride was added to each chamber and incubated for 2 min before measurements.

### Measurement of ENaC cleavage in HEK cells

The cell line HEK293-ENaC was a kind gift from Prof. Olivier Staub, University of Lausanne. These cells are stably transfected with αENaC, under the control of a glucocorticoid-inducible promoter, and β- and γ-ENaC expressed from a constitutive cytomegalovirus promoter^28^.

### Detection of MAp19 and MASP-2 in urine and plasma and purification of MASP-2 from serum

MAp19 and MASP-2 were measured in EDTA-plasma and urine with immunoassays described previously in detail^20,29^. MASP-2 associated with PRMs was purified from serum from healthy control persons by incubating serum diluted in TBS containing 10 mM Ca^2+^ with a mixture of Sepharose bead coated with acetylated-HSA (binding H-ficolin and L-ficolin) and Mannose-TSK beads (binding MBL).

### Mouse cortical collecting duct (mCCD) cells

mCCD cells (Clone 1, University of Lausanne) were cultured in growth media (DMEM/F12 1:1 (Invitrogen) supplemented with insulin (5 μg/ml), apo-transferrin (5 μg/ml), Na-selenate (60 nM), dexamethasone (50 nM), triiodothyronine(T3, 1 nM), penicillin (100 U/ml), streptomycin (130 μg/ml), EGF (10 ng/ml) and 2% FBS (Thermo Fischer Scientific)) at 37 °C in 5% CO_2_. Cells were used between passages 23–30. Permeable filter supports (pore size 0.4 μm, surface area 4.67 cm^2^; Transwell, Corning Costar) were coated with Collagen Type I extracted from rat tail (Millipore) and polymerized for 30 min at room temperature by adding 250 μl NH_3_aq (28%) in the empty compartments between the filters. The filters were incubated in growth media overnight at 37 °C in 5% CO_2_. mCCD cells were subcultured onto the filters and kept in growth media for 5 days, followed by culturing in the slightly modified growth media (DMEM/F12 1:1 with Insulin (5 μg/ml), Na-selenate (60 nM), Dexamethasone (3 nM), T3 (1 nM), penicillin (100 U/ml) and streptomycin (130 μg/ml) for 5 days. The media were changed every second day. Subsequently, the cells were incubated with experimental mCCD media (DMEM/F12 1:1 with penicillin (100 U/ml) and streptomycin (130 μg/ml) with or without 300 nM aldosterone (Merck) for 24 h.

### Polymerase chain reaction (PCR)

RNA from mCCD cells was extracted using a RNeasy mini kit (Qiagen). cDNA was synthesized with PrimeScript RT reagent Kit (Takara) at 37 °C 15 min, 85 °C 5 s, and 4 °C. Quantitative real-time (qRT)-PCR was performed using PowerUp SYBR Green Master Mix (Thermo Fisher scientific) by incubating 2 min at 50 °C, 2 min at 95 °C; followed by 40 cycles of: 95 °C 15 s, 60 °C 1 min before being held at 4 °C. Primers targeting MASP2 (5′-ACCGCTGCGAGTATGACTTT-3′, 5′-TGCATAGAAGGCCTCAAACC-3′) were applied. The primers does not target the splice varian MAp19. The data were analyzed by 7500 Fast Real-Time PCR systems with software v.2.04 (Applied Biosystems, Foster City, CA, USA). Human kidney and liver -tissue and mice tissue were RNA-extracted by phase separation using Trizol reagent (Merck). Turbo-DNAse free kit (Invitrogen) was applied, and cDNA was synthesized using the iScript cDNA Synthesis Kit (Bio-Rad). PCR amplification was performed with predesigned primer H_MASP2_3 (Sigma-Aldrich, RefSeq ID NM_006610) using PowerUp SYBR Green Master Mix (Thermo Fisher Scientific) at 95 °C for 3 min and 36 cycles of 95 °C 20 s, 60 °C 30 s, 72 °C 30 s, and 72 °C for 10 min before being held at 4 °C. The samples were separated on a 2% agarose gel with loading dye and a size marker (Sigma-Aldrich) at 125 V. H_2_O, and samples without reverse transcriptase, GADPH, and β-actin were included as controls.

### Statistics data access

Results were tested for normality using the Shapiro–Wilk normality test. If not normally distributed, log-transformation was performed, and data presented in a semi-logarithmic graph with geometric mean. Unpaired Student's t-test was used to test for differences. Correlations were investigated by linear regression. Graphs represent mean ± SD. P < 0.05 were considered significant.

## Results

### MASP-2 protein is present in human kidneys

**Figure 1:**
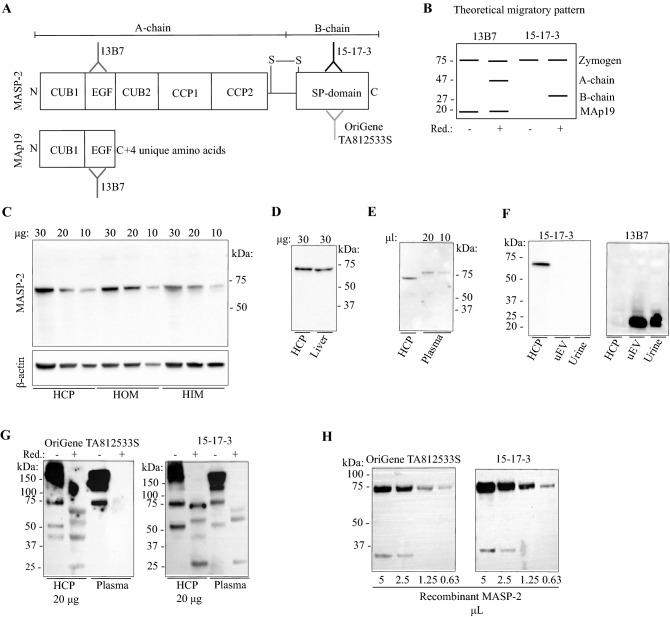
Detection of MASP-2 protein variants in liver, kidney and urine. (**A**) Three distinct murine monoclonal anti-human antibodies targeting MASP-2 were used, two reacting with epitopes within the SP-domain of MASP-2; an *in-house* (15-7-3) and a commercial (Origene), the third (13B7) reacting with an epitope in the N-terminal domain. The six domains of intact MASP-2 are illustrated, including the two CUB-domains, the epidermal growth factor (EGF)-like domain, the two complement control repeats (CCPs) and the serine protease (SP) domain. The alternatively spliced short variant MAp19 also shown. N- and C-terminals are outlined. (**B**) Predicted cleavage products detected by the two antibodies. Under non-reduced conditions, the antibody targeting the A-chain (13B7) would detect bands at ≈ 75 kDa (MASP-2) and ≈ 20 kDa (MAp19), and the antibody targeting the B-chain (15-7-3) would detect at ≈ 75 kDa. Under reduced conditions, 13B7 is expected to detect the 47 kDa A-chain if MASP-2 is activated, whereas 15-7-3 antibody is expected to detect the 27 kDa variant (the SP-domain) (Fig. 2B, lane 3 + 4). (**C**) When using the anti-SP-antibody (15-17-3), a band ≈ 70 kDa was detected in human kidney homogenate in both human pools of kidney cortex (HCP, n = 4), outer (HOM, n = 4) and inner medulla (HIM, n = 4) in reduced samples. (**D**). A similar band was detected in human liver homogenate, under reduced conditions, when using the anti-SP-antibody (15-17-3). HCP = human cortex pool (n = 4). (**E**) In human plasma, the anti-SP-antibody (15-17-3) detected a band migrating at ≈ 75 kDa. (**F**) Full length MASP-2 was not detected with protein from urinary extracellular vesicles (uEV) or in experimentally concentrated urine when using the 15-17-3 antibody. When testing the antibody targeting the A-chain (13B.7), no signal was detected in a kidney cortex pool (HCP, n = 4). In contrast, the alternative splice variant MAp19 was detected in uEVs and concentrated urine. (**G**) Under non-reduced condition, a band likely representing intact MASP-2 at 75 kDa was apparent in kidney cortex (HCP) and plasma with the in house 15-17-3 antibody whereas the commercial monoclonal antibody (OriGene #TA812533S) directed against the SP domain confirmed the presence of a 75 kDa band in non-reducing lanes. Both antibodies detected a band migrating slightly faster, at ≈ 70 kDa, in a human kidney cortex pool (HCP) and plasma. (**H**) Both the commercial antibody (OriGene #TA812533S) and the *in-house* monoclonal antibody (15-17-3) detected a band ≈ 75 kDa, when recombinant MASP-2 was applied to the membrane under non-reduced conditions. Original uncropped blots are presented in Supplementary Fig. S2A–F.

MASP-2 mRNA was not detected in human and murine kidney tissue while it was readily detected in human liver tissue (supplementary Fig. S1). To test if MASP-2 is present in the kidney at protein level. two different monoclonal anti-MASP-2 antibodies developed *in-house* (15-17-3 and 13B7) and a commercial antibody (Origene TA812533S) were used to detect MASP-2 protein in human kidney and liver homogenates, in plasma and in urinary extracelullar vesicles (uEVs) (Fig. 1A,B). Clone 15-17-3 targets the B-chain/serine protease (SP)-domain, whereas clone 13B.7 targets the A-chain of MASP-2, i.e., it reacts also with the alternative splice variant MAp19 (Fig. 1A,B). In the MASP-2 zymogen form, the A- and B-chains are connected via a peptide- and a disulfide bond (Fig. 1A,B). Once activated, it is only the disulfide bond that joins the A and B-chains. Thus, under reducing conditions, activated MASP-2 is predicted to migrate with separated A and B chains, as illustrated in Fig. 1B. In human kidney cortex tissue pool (n = 4), the SP domain-directed antibody detected a significant band migrating at ≈ 70 kDa in both inner- and outer medulla and cortex (Fig. 1C). β-actin was used as loading control. Semi-quantification for each kidney specific tissue showed a linear correlation between concentration and western blot signal (supplementary Fig. S2A) A similar ≈ 70 kDa band was detected in a human liver tissue pool (n = 4) (Fig. 1D). In plasma, the antibody reacted with a band that migrated at 75 kDa (Fig. 1E). Thus, full-length inactive MASP-2 that migrates with an apparent slightly lower molecular weight than in plasma is present in human kidney and liver tissue. By contrast, no full-length MASP-2 was detected in urinary extracellular vesicles (uEV) or experimentally concentrated crude urine (Fig. 1F). When using the antibody 137B, directed against the N-terminal domain, a ≈ 20 kDa band was detected in both urine and uEVs, corresponding to the alternative splice variant MAp19, while no signal for MAp19 was detected in human kidney cortex tissue (Fig. 1F). In urine and uEVs, the short isoform MAp19 is the dominant form. Because of the unexplained ~ 3–5 kDa difference in migration pattern between MASP-2 in plasma and fresh native tissue homogenates, specificity was further corroborated. Under non-reduced condition, the *in house* developed anti SP-domain antibody (15-17-3) resulted in a band in kidney cortex that migrated at 75 kDa and shifted to ~ 70 kDa under reduced conditions (Fig. 1G). A commercial antibody (OriGene #TA812533S) directed against the SP-domain confirmed the presence of a ≈ 75 kDa band under non-reduced conditions but did not seem to give clear signal in the reduced sample (Fig. 1G). In plasma, both antibodies recognized the zymogen form at 75 kDa (Fig. 1G). Other bands were exposed. Both anti-SP-antibodies recognized a band at ca 50 kDa and, at reducing conditions only, the beta-chain at ~ 25 kDa in tissue (Fig. 1G). We expect that the strong reactivity with bands at around 150–200 kDa in the non-reduced samples is due to reactivity with non-reduced IgG, i.e. the blot was developed with an anti-mouse IgG antibody, and some cross reactivity may be expected towards the human IgG on the blot. Both the commercial and the in-house antibody directed against the anti-SP domain detected a band ≈ 75 kDa, when a dilution series of pure human recombinant MASP-2 was present on the membrane (Fig. 1H).

**Figure 2:**
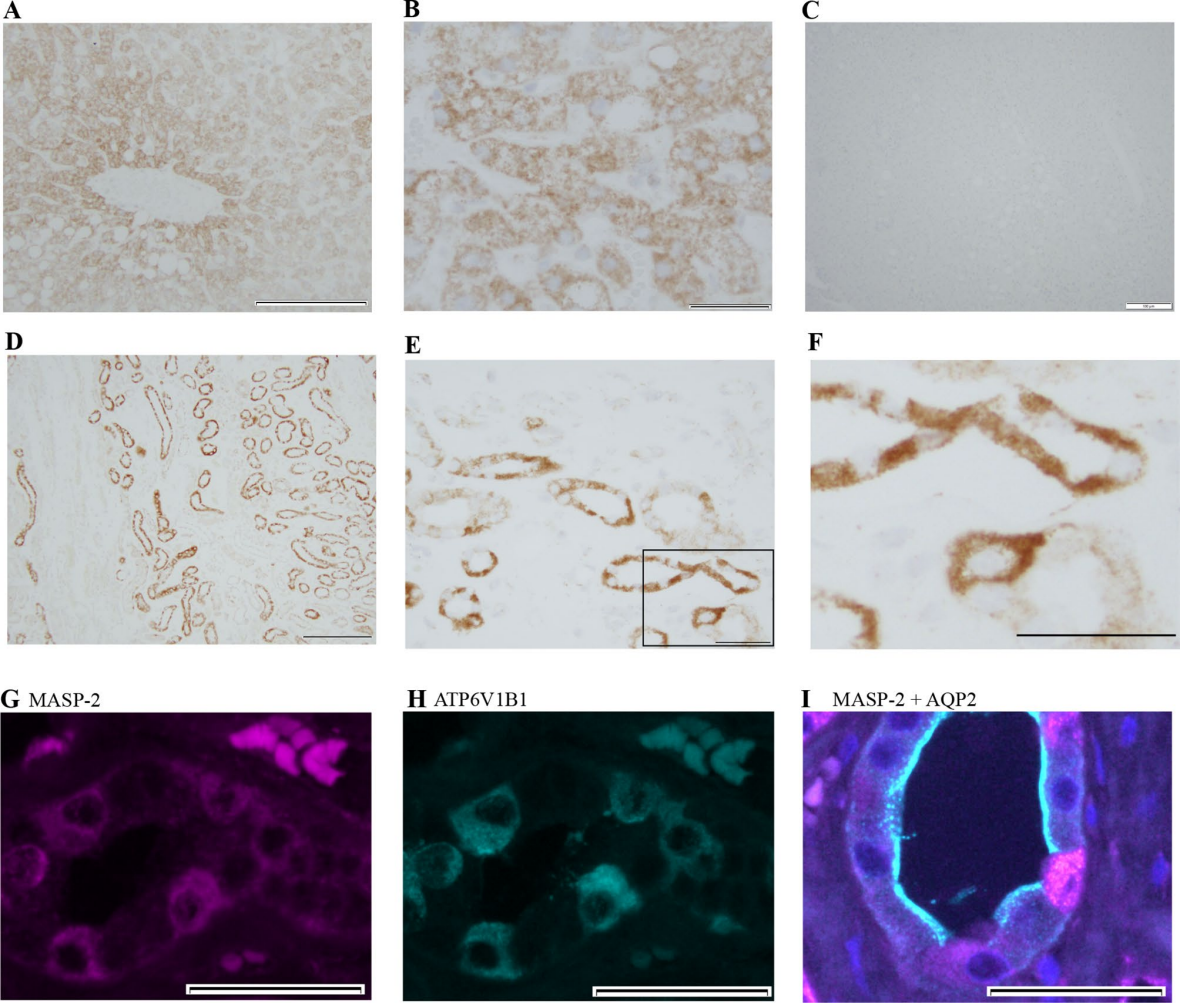
MASP-2 is localized in the intercalated cells of the collecting duct. (**A**,**B**) Using the antibody directed against the SP-domain of MASP-2 (15-17-3), immunohistochemical experiments revealed a positive signal in human liver sections that was associated with hepatocytes. (**C**) Negative control. (**D**–**F**) In human kidney sections, immunochemical signal from MASP-2 was detected in distinct tubular segments both in cortex and in medulla which were not proximal tubules. Along labeled segments, certain subsets of cells were not labeled (**E**,**F**). (**G**,**H**) MASP-2 co-localized with the intercalated cell marker ATP6V1B1. (**I**) MASP-2 did not co-localize with principal cell marker aquaporin-2 (AQP-2 turquoise, MASP-2 magenta, DAPI/nucleus = blue).

### MASP-2 immunoreactivity is present in intercalated cells in the human kidney collecting duct

The antibody 15-17-3 directed against the SP-domain (Fig. 1A) revealed immune-positive signals from human liver sections, where the signal was associated with hepatocyte cytoplasm with a tendency to stronger labeling of zone III cells pericentrally (Fig. 2A,B). No signal was detected in the absence of a primary antibody (Fig. 2C). In kidney, an immune-positive staining was detected in both cortex, inner and outer medulla. Three different mAbs targeting the SP-domain showed similar results. The commercial monoclonal antibody targeting MASP-2 yielded similar staining pattern (Supplementary Fig. S3). Thus, labeling signal for MASP2 was observed faintly in proximal convoluted tubules but not in glomeruli or in the vasculature (arteries and capillaries) (Fig. S3). MASP2 labeling was most prominent in the collecting ducts (Fig. 2D–F). More detailed analysis was performed in collecting ducts with special emphasis on principal cells, i.e. AQP2- positive cells, that express ENaC. In collecting ducts, the staining signal was localized mainly intracellularly, sparing the nucleus and with signal equally distributed at apical and basolateral sides (Fig. 2F). Confocal immunofluorescence detection confirmed MASP-2 localization in the collecting duct, where MASP-2 signal co-localized with the intercalated cell marker ATP6V1B1 (Fig. 2H,I). No co-localization with the principal cell-marker aquaporin-2 was detected (Fig. 2G). Thus, in the collecting ducts, MASP-2 is present in intercalated cells, and a direct intracellular interaction between ENaC and MASP2 is not likely under physiological conditions.

### Effect of sodium intake/circulating aldosterone on MASP-2 concentration in urine and plasma

**Figure 3:**
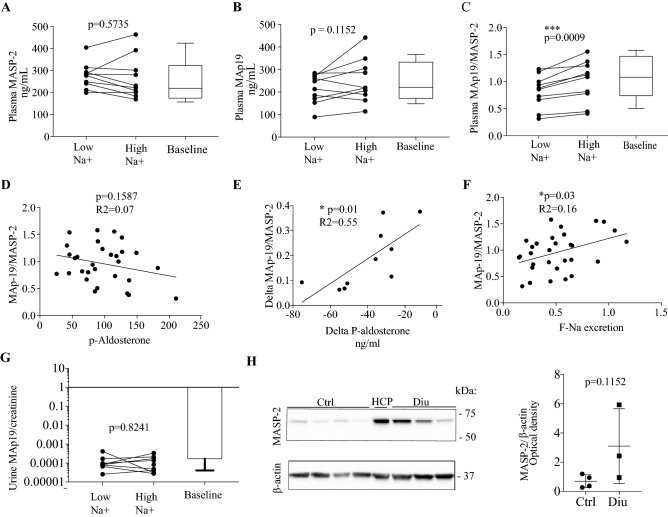
Changes in plasma concentration of MAp19 and MASP-2 in response to Na^+^ intake. (**A**,**B**) In a cohort of healthy young men (n = 10) ingesting a diet with high (250 mmol/day) and low (70 mmol/ day) Na^+^ content for five days, no difference was detected in plasma concentrations of MASP-2 and MAp19 in response to Na^+^ intake. Samples obtained on habitual diet were analyzed to allow comparison and increased power in correlation analysis (indicated as baseline). (**C**) The MAp19/MASP-2 ratio was significantly increased in conditions with high Na^+^ intake (p = 0.0009). (**D**,**E**) The ratio did not correlate with p-aldosterone (p = 0.16, R^2^ = 0.07), but the delta values of p-aldosterone in the individuals correlated significantly with the delta values of ratio MAp19/MASP-2 (p = 0.01, R2 = 0.55). (**F**) Ratio MAp19/MASP-2 correlated significantly with the fractional Na^+^ excretion (p = 0.03, R2 = 0.16). (**G**) No difference in urine MAp19/creatinine concentration ratio was detected with different Na + intake (p = 0.82). Intact MASP-2 was not detected in the urine. (**H**) In kidney cortex homogenate from patients receiving diuretics (Diu) before nephrectomy and therefore with predicted higher plasma aldosterone concentration than in patients not receiving medicine (Ctrl), no significant different in MASP-2 protein abundance was detected. Original uncropped blots are presented in Supplementary Fig. S2G. HCP = human kidney cortex pool, n = 4. p = plasma.

Next, we tested the hypothesis that MASP-2 secretion is dependent on aldosterone concentration in humans under normal physiological settings. By ELISA, there was no difference in plasma concentration of MASP-2 (Fig. 3A, p = 0.5735) or MAp19 (Fig. 3B, p = 0.1152) in healthy adult males receiving high (250 mmol/day) or low (70 mmol/day) Na^+^ diet. A low Na^+^ diet decreased significantly the plasma ratio MAp19/MASP-2 (Fig. 3C, p = 0.0009). The MAp19/MASP-2 ratio of the two isoforms produced from the MASP2 gene did not correlate with p-aldosterone concentration (Fig. 3D, p = 0.16, R^2^ = 0.07), but a significant correlation was detected between the delta values of p-aldosterone (Δp-aldosterone = p-aldosterone-high Na^+^ minus p-aldosterone-low Na^+^) (Fig. 3E, p = 0.01, R^2^ = 0.55). AA positive correlation was detected between plasma MAp19/MASP-2 ratio and the fractional Na^+^-excretion (Fig. 3F, p = 0.03, R^2^ = 0.16). In urine, no difference in MAp19/creatinine concentration ratio between the two diets was detected (Fig. 3G, p = 0.82). One test subject showed MAp19 below the detection limit in the urine samples from both high, low, and baseline samples, whereas another test person had MAp19 levels below detection range in the low Na^+^ period. The values below the detection range were not included in the statistical analysis. The ELISA assay did not detect any MASP-2 in crude or ex vivo experimentally concentrated urine. No correlation was detected between plasma aldosterone and MASP-2 concentrations (p = 0.13), MAp19 (p = 0.28) or urine MAp19 (p = 0.34) or between mean arterial blood pressure and MASP-2 (p = 0.11), plasma MAp19 (p = 0.14) or urine MAp19 (p = 0.20) (not shown). Furthermore, no correlation was detected between ratio MAp19/MASP-2 and uEV excretion of MAp19 (p = 0.10, R^2^ = 0.10) or MAp19 in the urine (p = 0.15, R^2^ = 0.09). There was a non-significant trend for more MASP-2 protein in kidney tissue from patients receiving diuretics (n = 3) compared with controls receiving no medicine (n = 4) (Fig. 3H, p = 0.1152).

### Effect of salt intake/plasma aldosterone on MAp19 protein abundance in uEVs

**Figure 4:**
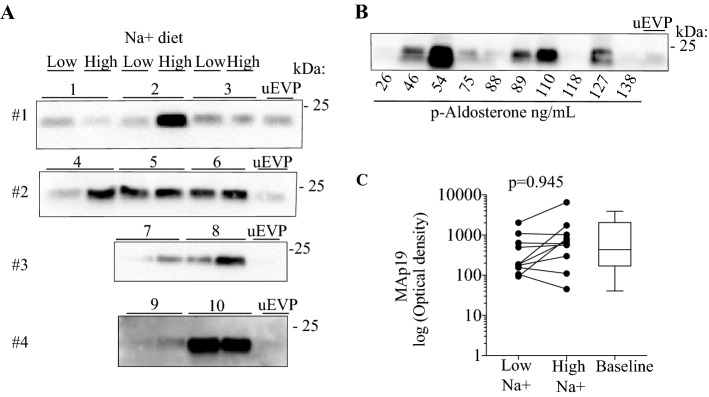
Effect of Na^+^intake on MAp19 in urine extracellular vesicles. (**A**) In a cohort of healthy young men receiving high (250 mmol/day) and low (70 mmol/day) Na^+^ diet, MAp19 was detected in all uEVs. Original uncropped blots are presented in Supplementary Fig. S1H. (**B**) No clear correlation between abundance of uEV-associated MAp19 protein and plasma aldosterone concentration was observed when samples were subjected to SDS-PAGE and immunoblotting according to plasma aldosterone concentration. Original uncropped blots are presented in Supplementary Fig. S1I. (**C**) There was no significant intra individual difference of uEV-associated MAp19 protein abundance between high and low Na^+^ intake (p = 0.09). The signal obtained with western blotting was normalized to the signal obtained from a pool of uEV (uEVP, n = 10) to allow comparison between blots.

To test for MASP-2-association with apical epithelial membranes along the tubular system, urine microvesicles were isolated and analyzed. Only MAp19 and not full-length MASP-2 was detected in uEVs (Fig. 4A,B). No significant relation with plasma aldosterone concentration was detected in baseline samples between individuals when ranging according to measured plasma aldosterone (Fig. 4B). In paired samples, no difference in MAp19 was detected between high and low dietary Na^+^ intakes (Fig. 4C).

### Co-expresseion of MASP-2 activates ENaC in vitro in *Xenopus* oocytes, but not in a stable cell line and no difference in γENaC cleavage products of is detected

**Figure 5:**
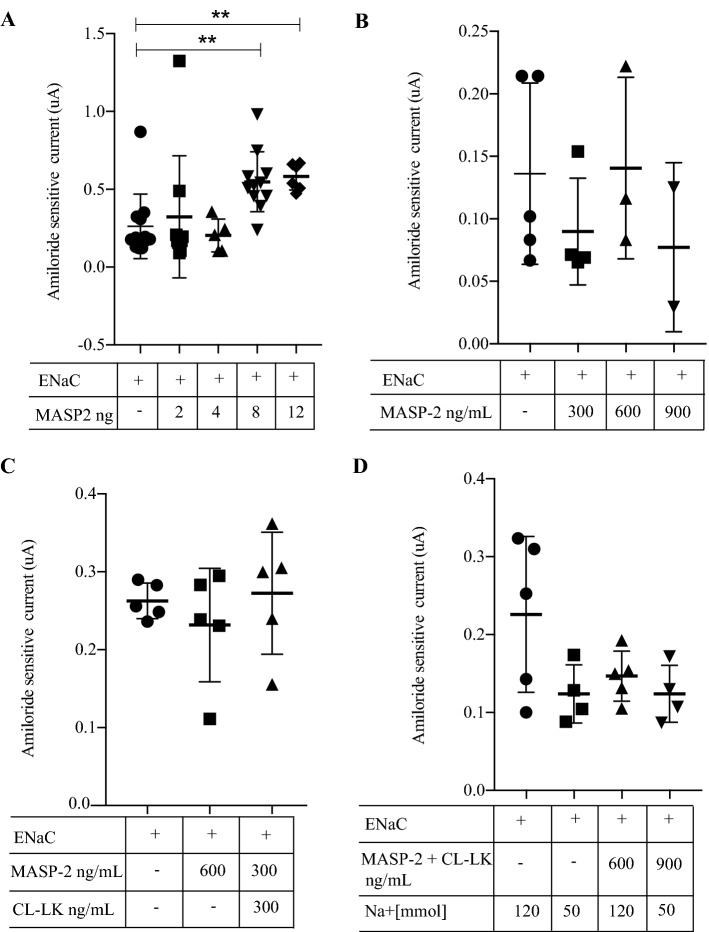
Effect of co-expression of MASP-2 and ENaC on current in Xenopus oocytes. (**A**) When co-injecting cRNAs for αβγENaC and MASP2, an increased amiloride-sensitive current was detected with a concentration of 8 and 12 ng MASP2. (**B**–**D**) In *Xenopus* oocytes injected with cRNAs encoding αβγENaC, no increased amiloride-sensitive current was detected when applying MASP-2 in the buffer, neither when adding the pattern recognition molecule collectin CL-LK (heteromer of collectin liver 1 and kidney 1), known to interact with MASP-2 (**C**), nor when decreasing the Na^+^ concentration of the buffer (**D**).

**Figure 6:**
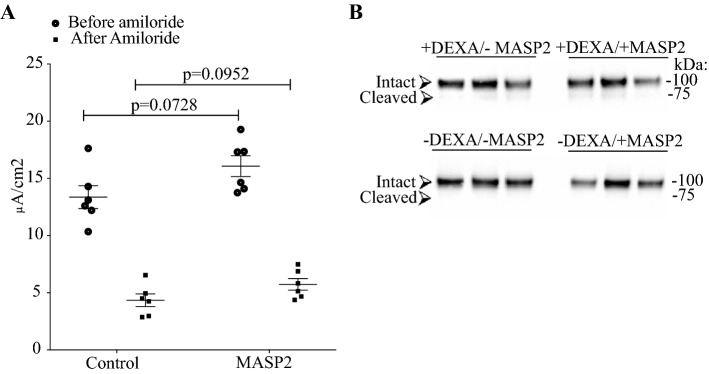
Transepithelial current responses in M1 cells transfected with MASP2. (**A**) No difference in Amiloride-sensitive current was detected between MASP-2 transfected and non-transfected M1 cells (n = 6 experiments for each condition). The time course of transepithelial- voltage, resistance or current for M1 cells transfected with MASP-2 and controls are shown in supplementary Fig. S4. (**B**) HEK293-cells with heterologous ENaC expression showed no difference in γENaC cleavage when subjected to MASP2. These cells constitutively express β- and γ-ENaC subunits, while the α-subunit is induced by dexamethasone (indicated as + DEXA). ENaC is, therefore, only surface localized in the presence of dexamethasone, and consistent with this, no γENaC cleavage was detected without dexamethasone. The predicted size of non-cleaved/intact and cleaved γENaC is indicated. Original uncropped blots are presented in Supplementary Fig. S2K.

To investigate if MASP-2 is able to activate ENaC, we used xenopus oocytes ENaC and MASP2 cRNA co-injected into *Xenopus* oocytes showed an increased amiloride-sensitive Na^+^ current at 8 ng and 12 ng of MASP-2 cRNA (Fig. 5A, p = 0.003). In contrast, incubating ENaC-cRNA injected oocytes with different concentrations of active MASP-2, no difference in amiloride-sensitive current was detected (Fig. 5B), neither when incubating with the associated pattern recognition molecule collectin CL-LK that forms complex with MASP-2 (Fig. 5C), nor when lowering the Na^+^ concentration of the buffer (Fig. 5D). To validate the above results another in-vitro experiment was performed, using monolayers of collecting duct normal or MASP2 stable transfected M1 cells cultured on filters. Subsequent western blotting was performed to assure transfection and only cells transfected with MASP-2 gave a signal (supplementary Fig. S4A). No differences in amiloride-sensitive current were detected between cells transfected with MASP-2, compared to non-transfected cells when performing EVOM measurements (Fig. 6A). Amiloride was added the last day of experiment (day 16). No different between the MASP-2 transfected cells and control cells was detected in voltage, current or resistance at any point of the experiement (supplementary Fig. S4B–D). To assess MASP2-induced γENaC cleavage, we used HEK293-cells with constitutive β- and γ-ENaC subunits expression and dexamethasone-inducible α-subunit expression. In these cells, ENaC is only surface localized in the presence of dexamethasone^28^, and consistent with this, no γENaC cleavage was detected without dexamethasone (Fig. 6B). Incubation with soluble MASP2 protein did not affect ENaC cleavage compared to control in dexamethasone-treated cells (Fig. 6B).

## Discussion

The present set of data show the presence of MASP-2 protein in human kidney collecting duct intercalated cells despite undetectable kidney MASP2 mRNA expression. MASP-2 can activate ENaC in vitro in *Xenopus* oocytes, while stable expression of MASP-2 in M-1 collecting ducts cells did not affect amiloride-sensitive transepithelial current, and no difference in ENaC-cleavage is revealed. In healthy young male adults, there were no changes in MASP-2 in plasma or urine that related to changes in sodium intake and aldosterone plasma concentration. There was an increased MAp19/MASP-2 ratio in plasma with high Na^+^-diet suggesting altered regulation of mRNA splicing. Taken together, MASP-2 is not likely to exert aldosterone-driven, luminal, physiological activation of ENaC by proteolytic cleavage of the γ-subunit.

In the present study, we showed that MASP-2 can activate ENaC in vitro when co-expressed in *Xenopus* oocytes, but not in MASP2 transfected M1 cells. On the other hand, soluble MASP2 did not activate ENaC in oocyte or cause γENaC cleavage in ENaC expressing HEK293. The reason for this difference is not known, but together this indicates that the role of MASP-2 in the kidney is less likely to involve ENaC regulation. In line with this, several other proteases have been shown to activate ENaC in vitro, including prostasin^4,9,30,31^, tissue-kallikrein^32^, plasma-kallikrein^8^ and TMPRSS4^9,33^. However, when tested in vivo, typically by gene targeting of the proteases, no phenotypes were detected^7,8,32^.

Since the surface binding of circulating MASPs requires specific interaction partners, we speculated that collectin CL-LK could be the link between ENaC and MASP-2 since it binds to mannose and l-fucose-rich structures and ENaC is known to be highly glycosylated when expressed on the surface^34^. CL-LK is a soluble C-type lectin produced by several non-immune cells, including epithelial cells of the renal tract^35^. However, activation was not observed when co-incubating soluble MASP-2 alone or in complex with collectin CL-LK, which is known to avidly form complex with MASP-2 and activate the complement system. CL-K1 binding activity is also strongly and positively modulated by low concentrations of Na^+^^36^, but no difference was detected by lowering Na^+^ concentration.

Previous studies showed that MASP2 mRNA was almost exclusively expressed in the liver^19,20,37^, which was confirmed in the present study, where no mRNA expression of MASP2 was detected in neither mice or humans. It is puzzling how MASP-2 protein enters the tubular epithelium. One study suggests that MASP-2 is filtered across the glomerular barrier despite its size^38^; however, MASPP-2 was not detected in urine or uEVs (MAp19 only). The finding of intact MASP-2 protein in the kidney is consistent with two previous studies showing diffuse but strong immunopositive signal in the kidney^20,39^. The MASP-2 detected in kidney by western blotting is around 5 kDa below the MASP-2 detected in plasma. The molecular weight in kidney was confirmed using two independent monoclonal antibodies. We speculate that the difference in migration could be due to the active form of purified MASP-2 migrates slower on western blotting compared to the zymogen, which has previously been the case for the comparable MASP-1^40^. In other words, it is the zymogen form of MASP-2 which appears to be present in the kidney tissue. In the present study, MASP-2 immunoreactivity was associated with the intercalated cells in the collecting duct and not in ENaC expressing AQP2-positive principal cells. Thus, a direct intracellular interaction between ENaC and MASP2 is not likely under physiological conditions. Previous studies have suggested that the intercalated cells play a role in the innate immune system^41^; however, the role of MASP-2 in intercalated cells is unknown. The mechanism for selective accumulation of zymogen MASP-2 in intercalated cells is not clear.

Another interesting finding was that full length MASP-2 was not detected in urine and uEVs in seemingly contrast to previous studies^14,18^. Since the referred studies utilized mass spectrometry-based proteome analysis of urine, we speculate that the detected MASP-2 in these studies based on peptide patterns was the short variant MAp19. The consistent detection of MAp19 in both uEVs and urine by redundant immunoblotting and ELISA further supports this interpretation. With a molecular size below 25 kDa a fraction in plasma would be filtered across the glomerular barrier. The previous study that reported significant regulation of MASP-2 by aldosterone^14^ did not reveal the peptide sequence used. When looking at the study by Damkjær et al*.*, the peptide sequences used target MASP-2 at amino acids 21–56, meaning that it is likely the alternative splice variant MAp19^18^. Nevertheless, a study using activity-based protein profiling showed that MASP-2 was present in an active form in the urine^17^ and a recent study identified the individual proteases in urine from healthy and nephrotic humans and found MASP-2 to be one of the most abundant proteases in urine from healthy humans^42^.

We did not detect regulation of MASP-2 concentration by aldosterone. The plasma ratio of MAp19/MASP-2 was significantly decreased with low Na^+^ intake, which could indicate that the MASP-2 splice variant's transcription is favored towards MAp19 in this condition. The plasma MAp19/MASP-2 ratio correlated significantly with change in plasma aldosterone concentration, which is compatible with a causal relation. We speculate that aldosterone could regulate the transepithelial transport of MASP-2 and/or MAp19, as seen with other proteins like albumin^43^. A previous study found no correlation between plasma MAp19 and urine excretion of MAp19, indicating that glomerular filtration couldn’t fully account for urine MAp19^20^. The lack of significant difference in the kidney tissue from patients receiving diuretics could be due to the small number of included patients and the use of medication which changes aldosterone (ACEi, AT1 blockers).

The physiological function of MASP-2 in the kidney is not clarified. MASP-2 has an established role in innate immunity and in certain pathophysiological conditions, it may promote kidney injury. Thus, absence of MASP-2 is protective in a transplant model of renal ischemia–reperfusion injury in mice^44^. The absence of MASP-2 is also protective in protein overload nephropathy^45^. MASP-2 contributes to IgA nephropathy, and an antibody against MASP-2 to treat IgA nephropathy (OMS721, Omeros, Seattle, WA, USA) is currently under investigation, where the phase-2 trial showed reduced proteinuria in IgA nephropathy by 50–90% and stabilized or increased glomerular filtration rates when treating with the antibody (Clinical Trial ID: NCT02682407)^46^.

In conclusion, full-length catalytically active MASP-2 can activate ENaC current in vitro when co-expressed in some experimental settings but does not appear to be co-expressed or co-localized with ENaC in the principal cells in human, is not present in intact form in urine and does not lead to ENaC cleavage ex vivo and is not regulated by aldosterone. Together, this indicates that MASP-2 is less likely to mediate physiological, proteolytic regulation of ENaC. The short MASP-2 isoform MAb19 is present in soluble and membrane bound form in urine and the ratio of MAp19 and MASP-2 changed with aldosterone. Its significance remains to be determined.

## Supplementary Information

Supplementary Figures.

## Data Availability

The datasets generated and/or analysed during the current study are available in the figshare repository, 10.6084/m9.figshare.19719208.
