# Supplementary material for: Mannan-binding lectin serine protease-2 (MASP-2) in human kidney and its relevance for proteolytic activation of the epithelial sodium channel
Source: Sci Rep. 2022 Sep 24;12:15955. doi: 10.1038/s41598-022-20213-8 (PMC9509361; doi:10.1038/s41598-022-20213-8)
Supplement: Supplementary file 1 — Supplementary Figures. [file 41598_2022_20213_MOESM1_ESM.pdf]

# Supplementary figures

## Mannan-binding lectin serine protease-2 (MASP-2) in human kidney and its relevance for proteolytic activation of the Epithelial Sodium Channel

Rikke Zachar*[1], Steffen Thiel[2], Søren Hansen[3], Maiken Lumby Henriksen[3], Mikkel-Ole Skjoedt[4], Karsten Skjodt[3], Zohra Hamzaei[1], Kirsten Madsen[1,5] , Lars Lund[6], Edith Hummler[7], Per Svenningsen[1], Boye Lagerbon Jensen[1, 6]

Department of [1]Cardiovascular and Renal Research, Institute of Molecular Medicine, University of Southern Denmark, Odense, Denmark, [2]Department of Biomedicine, Aarhus University, Denmark, [3] Department of Cancer and Inflammation, Institute of Molecular Medicine, University of Southern Denmark, Odense, Denmark; [4]Department of Clinical Immunology, Copenhagen University Hospital, Denmark; Departments of [5]Pathology and [6]Urology and Clinical Department, Odense University Hospital, Odense, Denmark, [7]Department of Biomedical Sciences, University of Lausanne, Switzerland. *Corresponding author

**Figure legends**

*S1. PCR*

A) MASP-2 mRNA was undetectable in mouse kidney, while housekeeping control gene transcripts (β-actin and GAPDH) yielded the expected amplification products with kidney cDNA as template. B) With mouse liver cDNA as template, the MASP-2 mRNA was readily detected, while no MASP-2 mRNA was detected in kidney. Uncropped gel shown below, box indicates the cropped version C) We speculated if MASP-2 expression was dependent on aldosterone concentration, however murine CCD cells with and without aldosterone stimulation failed to show MASP-2 mRNA expression. Mouse-liver was used as positive control, and set to 1000 % to detect even small amount of MASP2. The signal was normalized to beta-actin D) MASP2 mRNA was detected in pooled human liver tissue total RNA (n=4), but not in pooled human kidney tissue neither in the outer / inner medulla nor in the cortex.

*S2. Uncropped western blotting gels*

A) Uncropped version of figure 1C, with and without merged marker respectively. HCP = human cortex pool. HOM = human outer medulla. HIM = human inner medulla. Exposure time as indicated. (MASP-2) and 4 sec (beta-actin).Semi-quantification is presented in the graph. B) Uncropped version of figure 1D, with and without merged marker respectively. Exposure time 100 sec. A negative control and HEK-cells expressing MASP2 was part of the original blot, these were not included, since they did not added further value to the data set. C) Uncropped version of figure 1E, with and without merged marker respectively. D) Uncropped version of figure 1F, with and without merged marker respectively. Exposure time as indicated. HEK-cells expressing MASP2 was part of the original blot, these were not included, since they did not added further value to the data set. E) Merged marker of figure 1G. F) Uncropped version of figure 1H, with merged marker. Exposure time as indicated. G) Uncropped version of figure 3H with and without merged marker. Exposure time as indicated. The red color is due to over-exposure. H) Uncropped version of figure 4A. Number of test subject indicated below. To allow comparison between blots a urinary extracellular vesicle pool (uEVP) was included in each blot. I) Uncropped version of figure 4B. Aldosterone concentration as indicated. J) Uncropped blots with merged marker of figure 6B, with and witout merged marker. Exposure time as indicated. Red boxes indicate regions of the original blots used in main figures.

*S3.*

A, B, C) The commercial monoclonal antibody targeting MASP-2 (OriGene #TA812533S) showed labeling in kidney tubuli in three different human kidney tissue samples. D) Negative control without primary antibody. *DOI:* 10.6084/m9.figshare.19174952

*S4. EVOM measurements M1cells.*

A) Western blot control of M1 cell transfected with MASP2 and negative controls. Full length blot is shown below, exposure time 10 sec, exposure time 300 sec and merged marker respectively. The three first rows all represent human kidney cortex pool (HCP). Row 2 and 3 were an attempt to make deglycosylation, but subsequent analyzes showed that this was not succeced, why the figure was not included. Recombinant MASP-2 as well as supernatant (SN) from cells expression MASP-1, 2 and 3 respectively were included, but since these data did not added further value to the data set. they were not included. B, C, D) No difference in voltage (B), current (C) or resistenace (D) were detected between MASP-2 transfected and non-transfected M1 cells grown on permeable filters. Days indicate days from transfection. The experiments were performed on six individuals cell chambers for each conditions. Results are shown as +/- SD.

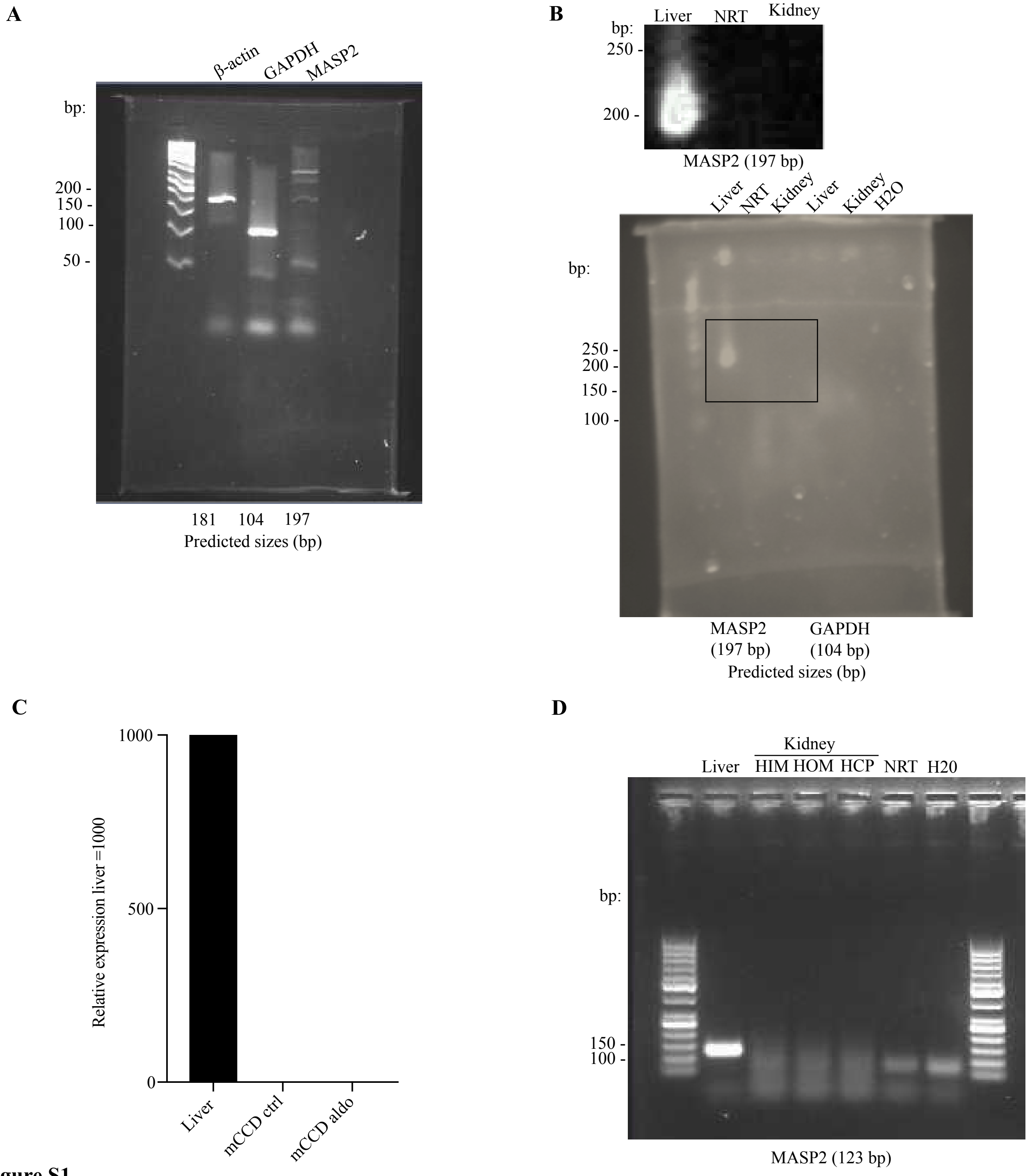
A
β-actin
GAPDH
MASP2
bp:
200 -
150 -
100 -
50 -
181
104
197
Predicted sizes (bp)
B
Liver
NRT
Kidney
bp:
250 -
200 -
MASP2 (197 bp)
Liver
NRT
Kidney
Liver
Kidney
H2O
bp:
250 -
200 -
150 -
100 -
MASP2
(197 bp)
GAPDH
(104 bp)
Predicted sizes (bp)
C
1000
500
0
Relative expression liver =1000
Liver
mCCD ctrl
mCCD aldo
D
Kidney
Liver
HIM
HOM
HCP
NRT
H20
bp:
150 -
100 -
MASP2 (123 bp)

**Figure S1**

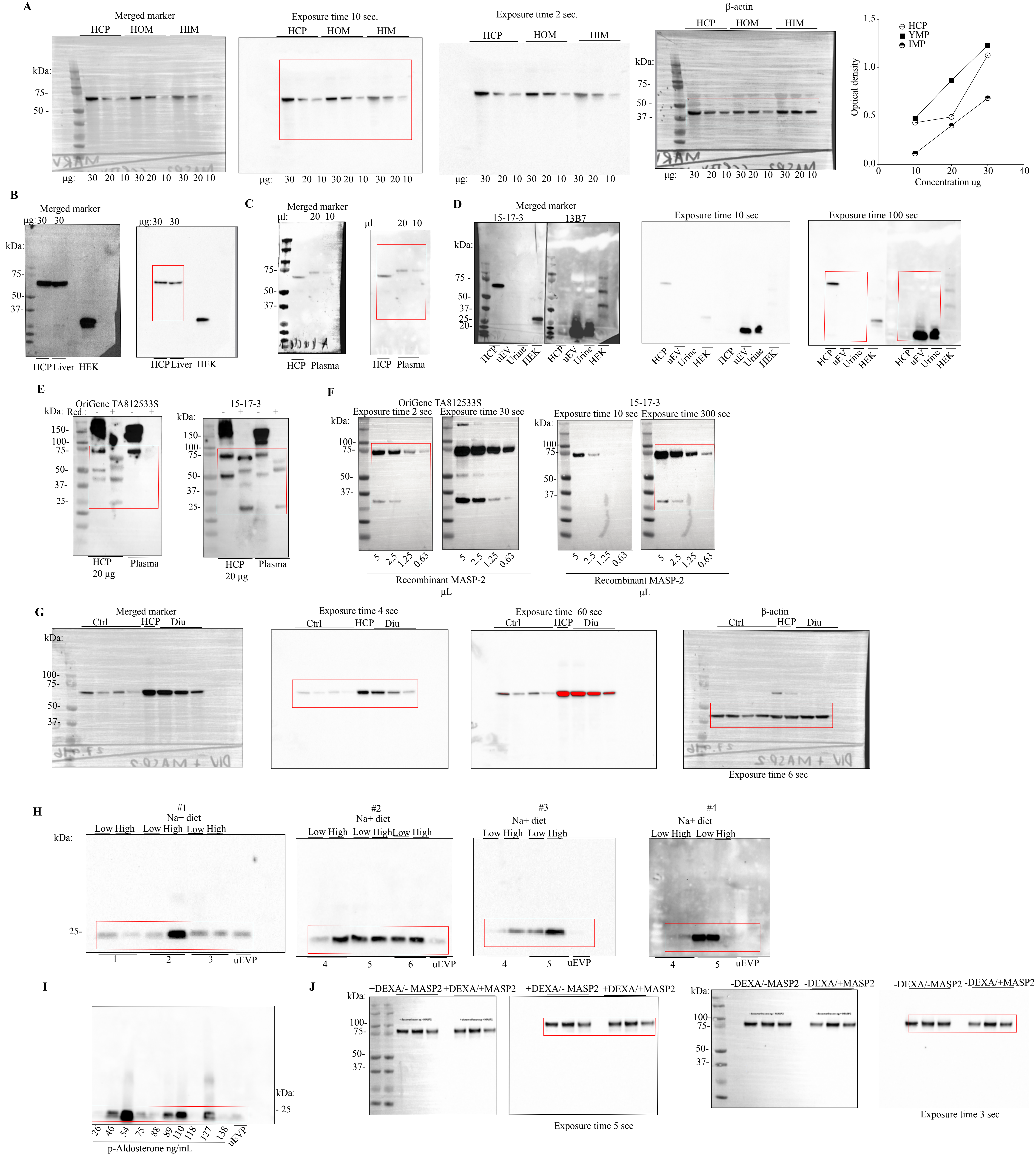
A
Merged marker
HCP HOM HIM
kDa:
75-
50 -
µg: 30 20 10 30 20 10 30 20 10
Exposure time 10 sec.
Exposure time 2 sec.
β-actin
37 -
HCP
YMP
IMP
Optical density
Concentration ug
B
Merged marker
µg:30 30
37-
HCP Liver HEK
C
µl: 20 10
HCP Plasma
D
15-17-3
13B7
25-
20-
HCP uEV Urine HEK
Exposure time 10 sec
Exposure time 100 sec
E
OriGene TA812533S
15-17-3
Red.: - + - +
150-
100-
HCP 20 µg
Plasma
F
Exposure time 2 sec
Exposure time 30 sec
Exposure time 300 sec
5 2.5 1.25 0.63
Recombinant MASP-2
µL
G
Ctrl HCP Diu
Exposure time 4 sec
Exposure time 60 sec
Exposure time 6 sec
H
#1
#2
#3
#4
Na+ diet
Low High
uEVP
I
p-Aldosterone ng/mL
26 46 54 75 88 89 110 118 127 138 uEVP
J
+DEXA/- MASP2
+DEXA/+MASP2
-DEXA/-MASP2
-DEXA/+MASP2
Exposure time 5 sec
Exposure time 3 sec

**Figure S2**

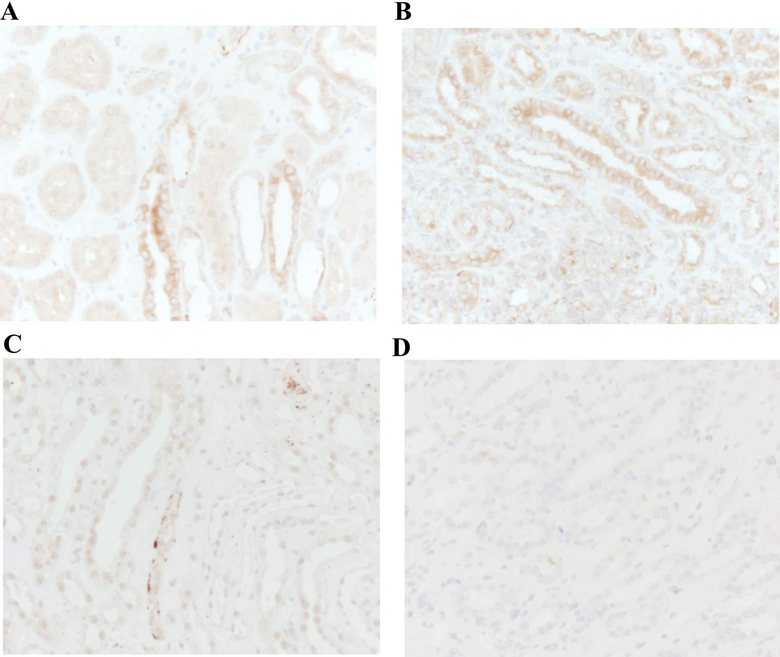


**Figure S3**

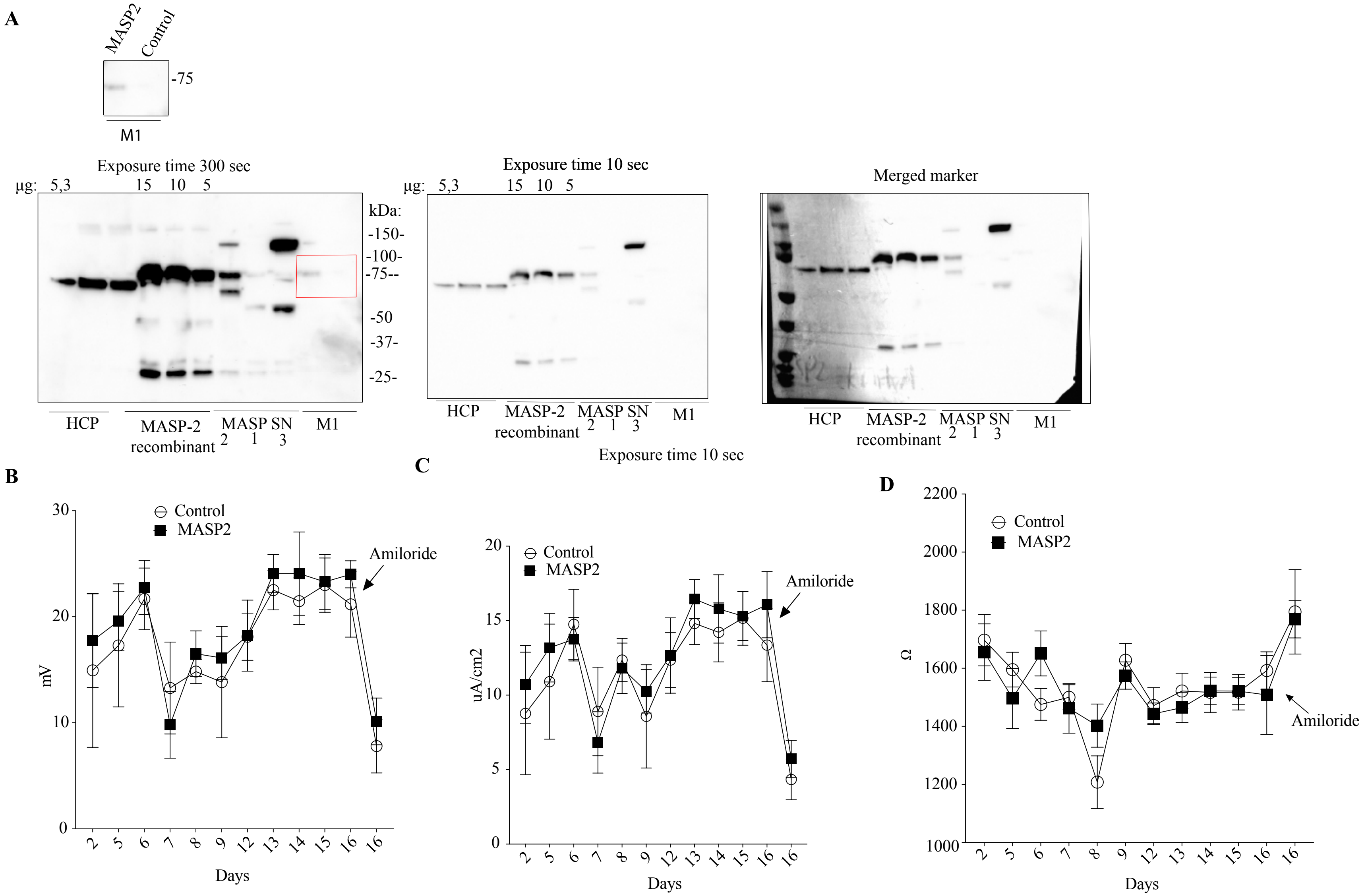

A
MASP2
Control
-75
M1
Exposure time 300 sec
µg: 5,3 15 10 5
kDa:
-150-
-100-
-75--
-50
-37-
-25-
HCP
MASP-2 recombinant
MASP SN 2 1 3
M1
Exposure time 10 sec
µg: 5,3 15 10 5
HCP
MASP-2 recombinant
MASP SN 2 1 3
M1
Merged marker
HCP
MASP-2 recombinant
MASP SN 2 1 3
M1
Exposure time 10 sec
B
Control
MASP2
Amiloride
mV
Days
C
Control
MASP2
Amiloride
uA/cm2
Days
D
Control
MASP2
Amiloride
Ω
Days


**Figure S4**
